# Relationship between circulating vascular endothelial growth factor and its soluble receptor in patients with hemorrhagic fever with renal syndrome

**DOI:** 10.1038/s41426-018-0090-5

**Published:** 2018-05-16

**Authors:** Emil Pal, Misa Korva, Katarina Resman Rus, Nataša Kejžar, Petra Bogovič, Franc Strle, Tatjana Avšič-Županc

**Affiliations:** 1 Department of Infectious Diseases, Murska Sobota General Hospital, 9000 Murska Sobota, Slovenia; 2 Institute of Microbiology and Immunology, Faculty of Medicine, University of Ljubljana, 1000 Ljubljana, Slovenia; 3 Institute for Biostatistics and Medical Informatics, Faculty of Medicine, University of Ljubljana, 1000 Ljubljana, Slovenia; 4 Department of Infectious Diseases, University Medical Centre Ljubljana, 1000 Ljubljana, Slovenia

## Abstract

Hemorrhagic fever with renal syndrome (HFRS) is characterized by endothelial dysfunction with capillary leakage without obvious cytopathology in the capillary endothelium. The aim of the study was to analyze the kinetics of vascular endothelial growth factor (VEGF) and its soluble receptor (sVEGFR-2) in HFRS patients infected with Dobrava (DOBV) or Puumala virus (PUUV). VEGF and sVEGFR-2 levels were measured in daily plasma and urine samples of 73 patients with HFRS (58 with PUUV, 15 with DOBV) and evaluated in relation to clinical and laboratory variables. In comparison with the healthy controls, initial samples (obtained in the first week of illness) from patients with HFRS had higher plasma and urine VEGF levels, whereas sVEGFR-2 levels were lower in plasma but higher in urine. VEGF levels did not differ in relation to hantavirus species, viral load, or the severity of HFRS. The comparison of VEGF dynamics in plasma and urine showed the pronounced secretion of VEGF in urine. Significant correlations were found between daily VEGF/sVEGFR-2 levels and platelet counts, as well as with diuresis: the correlations were positive for plasma VEGF/sVEGFR-2 levels and negative for urine levels. In addition, patients with hemorrhagic manifestations had very high plasma and urine VEGF, together with high urine sVEGFR-2. Measuring the local secretion of sVEGFR-2 in urine might be a useful biomarker for identifying HFRS patients who will progress to severe disease.

## Introduction

Pathogenic hantaviruses are etiologic agents of two clinical syndromes in humans, namely, hemorrhagic fever with renal syndrome (HFRS) in Eurasia and hantavirus cardiopulmonary syndrome (HCPS) in the Americas^1^. Hantavirus disease is a systemic illness targeting different organs and organ systems^2^. The clinical spectrum ranges from asymptomatic infection to a severe course with fatal outcomes, depending, in part, on the causative virus^3–5^. Endothelial dysfunction with temporary capillary leakage is the hallmark of the disease, resulting in tissue edema and organ failure, although the capillary endothelium displays no obvious cytopathology^3,6–9^. The vascular leakage is likely to be a multifactorial process, influenced by virus characteristics, viral load, and host factors^2,4^. Cytokines and chemokines are involved in the pathogenesis of disease and correlate with disease progress and outcome^3,6,10^. Cytokines may play more than one role by exerting various functions in a local and time-dependent manner; such multifactorial function might explain why different studies have shown the diverse impacts of cytokines on disease outcome^3,11,12^.

One such elusive cytokine, assessed in several studies, is a vascular endothelial growth factor (VEGF) and its soluble receptors^8,13–21^. VEGF is a family of five proteins, VEGF-A, VEGF-B, VEGF-C, VEGF-D, and placental growth factor (PlGF), which are coded on separate genes in humans. VEGF-A can be generated by almost all cells under hypoxic or other stress conditions, including endothelial cells; its most distinctive activity is the ability to render microvessels hyperpermeable^22^. In doing so, VEGF-A initiates a cascade of events that result in extravasation of plasma and plasma proteins, edema, clotting, and deposition of a provisional fibrin stroma that serves as a template for fibroblast and endothelial cell migration, leading to the formation of scar tissue^23^. VEGF acts locally (within 0.5 mm of release) through binding to the cognate receptors and applies downstream signaling through multiple signaling pathways that differ in their time courses^22,23^. VEGF receptors VEGFR-1 and VEGFR-2 are high-affinity transmembrane tyrosine kinase receptors that are preferentially expressed in proliferating endothelial cells and regulate proliferation and migration of the cells by binding circulating VEGF. Soluble VEGFR-2 receptor is a sponge that soaks up and inactivates VEGF and normally prevents generalized vascular permeability^24,25^.

Several studies have shown involvement of VEGF/sVEGFR-2 in endothelial activation during the febrile stage of hantavirus disease and an association with disease severity^14–17,19–21^. Pathogenic hantaviruses bind to αvβ3 integrin and increase the vascular permeability of endothelial cells in response to VEGF through phosphorylation and internalization of vascular endothelial cadherin and the ensuing disassembly of adherens junctions on the intracellular cleft^26–29^. However, some more recent studies point to the role of VEGF in endothelial remodeling and repair rather than dysfunction or damage^7,8,13^. In patients with HCPS, VEGF levels in serum are normal, whereas they are elevated in pulmonary edema fluid and activated pulmonary peripheral blood monocytes, suggesting localized rather than systemic excretion of VEGF^13^.

The aim of our study was to describe the kinetics of VEGF and sVEGFR-2 in plasma and urine samples obtained from HFRS patients infected with PUUV or DOBV. Levels of VEGF and sVEGFR-2 in plasma and urine samples were compared with viral load and selected clinical and laboratory parameters to evaluate the usefulness of VEGF and sVEGFR-2 as biomarkers for disease severity.

## Results

### VEGF and sVEGFR-2 in initial plasma and urine samples in relation to hantavirus species

**Fig. 1:**
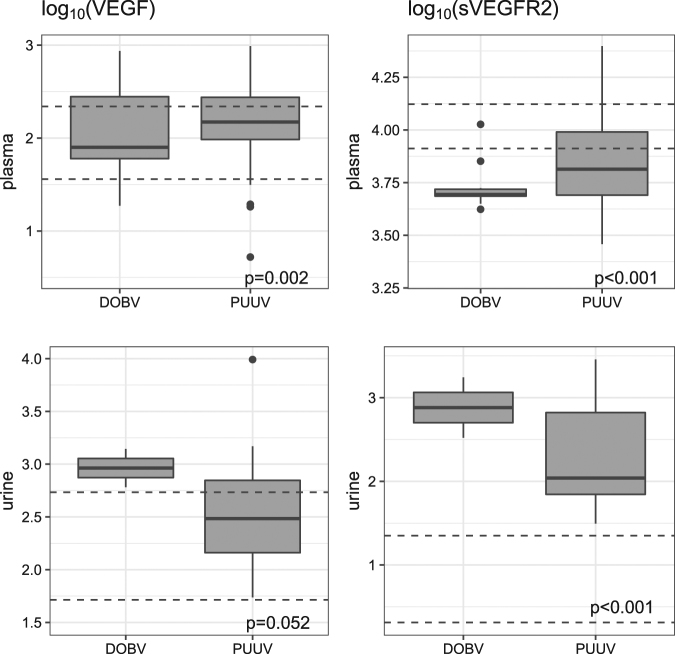
Comparison of VEGF and sVEGFR-2 levels in initial plasma and urine samples in HFRS patients. VEGF (LEFT) and sVEGFR-2 (RIGHT) were measured in samples obtained in the first week of illness (initial samples). UP: plasma samples; DOWN: urine samples. VEGF and sVEGFR-2 levels are shown as log_10_ pg/ml. Dashed lines indicate 95% confidence interval levels in the control group. The statistical comparison was made for all patients (regardless of virus) vs. controls

VEGF levels in the initial plasma samples were significantly higher in HFRS patients than in the control group (Fig. 1), but no difference was observed in relation to hantavirus species or severity of the disease course (Table 1). In contrast to VEGF, levels of sVEGFR-2 in initial plasma samples were significantly lower in patients with HFRS than in the control group; the same trend was observed in patients with severe PUUV infection (Table 1). In the initial urine samples, both VEGF and sVEGFR-2 were higher in HFRS patients than in the control group, but the difference was significant only for sVEGFR-2 (Table 1).

**Table 1:** VEGF and sVEGFR-2 levels in plasma and urine samples in HFRS patients

			No. of patients	VEGF		sVEGFR-2	
				Median (min–max) [pg/ml]	*p*	Median (min–max) [pg/ml]	*p*
Plasma samples							
Control group			51	**107.1** (35.9−262.8)	**0.008**	**10326** (7800−14,662) **5992** (2872−25,000)	**<0.001**
HFRS patients			68^a^	**147.7** (5.3−976.8)			
DOBV		All	12	79.8 (18.7−866.0)	NT	4943 (4205−10,641)	NT
		Severe	7	73.6 (18.7−490.0)		4997 (4843−10,641)	
		Mild	5	265.3 (60.2−866.0)		4676 (4205−7113)	
PUUV		All	56	149.1 (5.3−976.8)		6518 (2872−25,000)	
		Severe	15	141.4 (31.4−601.6)	0.172	**4311** (3200−20,117)	**<0.001**
		Mild	41	149.1 (5.3−976.3)		7014 (2872−25,000)	
Urine samples							
Control group			31	241.3 (20.5−657.7)	0.068	**14** (0−25)	**<0.001**
HFRS patients			21	437.9 (54.6−9821.0)		**100** (0−2864)	
DOBV		All			NT		
		Severe	2	1001 (605.0−1397)		1042 (332−1752)	NT
		Mild					
PUUV		All	19	304.6 (54.6−9821)		80 (0−2864)	
		Severe	4	1241 (266.0−9821)	NT	**1116** (63−2864)	**<0.001**
		Mild	15	182.1 (54.6−1282)		79 (0−1844)	

The initial sample was obtained in the first week of illness. *p* values indicate comparison between HFRS patients and the control group. PUUV-infected patients with severe disease progression were also compared with the control group

*NT* not tested statistically. The bold numbers are statistically significant.

^a^ Two PUUV and three DOBV-infected HFRS patients were hospitalized later in the disease course

### Association between viral load and plasma VEGF and sVEGFR-2

Mixed effect regression models were used to compare VEGF and sVEGFR-2 levels and viral load on consecutive days. A significant association was confirmed between sVEGFR-2 and viral load (*p* = 0.007), where an increase of viral load correlated with decreased plasma sVEGFR-2. Regarding plasma VEGF and viral load, a possible nonlinear association was found only for DOBV-infected patients (*p* = 0.018).

### Association between VEGF/sVEGFR-2 and clinical or laboratory parameters

Analysis of association between time-dependent secretion of plasma VEGF levels (transformed via log_10_) and diuresis, eGFR, creatinine (log_10_), platelet count, CRP, procalcitonin (log_10_), and D-dimer showed a significant positive linear association between plasma VEGF and platelet count and diuresis (Table 2) and a non-linear positive association between plasma VEGF and CRP levels (*p* = 0.005).

**Table 2:** Association between VEGF/sVEGFR-2 levels and diuresis/platelet count in serially measured plasma and urine samples from patients with HFRS

	**PLASMA**		**URINE**	
	log_10_ VEGF	log_10_ sVEGFR-2	log_10_ VEGF	log_10_ sVEGFR-2
Diuresis [ml]	0.00004 (*p* < 0.001)	0.0004 (*p* < 0.001)	−0.0001 (*p* < 0.001)	−0.0001 (*p* = 0.001)
Platelet count [10^9^/l]	0.0009 (*p* < 0.001)	0.00004 (*p* < 0.001)	−0.002 (*p* = 0.001)	−0.004 (*p* < 0.001)

Estimated mixed effect regression coefficients for tested parameters with *p* values are shown. Daily diuresis was measured in 58 HFRS patients and daily platelet count was measured in 71 HFRS patients. For urine samples, mixed effect regression coefficients were tested in 18 patients for diuresis and 21 patients for platelet count

For plasma sVEGFR-2, only associations with platelet count and daily diuresis were tested: significant positive linear associations with both platelet count and diuresis were found (Table 2). The latter was also found in a subgroup of PUUV-infected patients (*p* < 0.001).

In urine, both VEGF and sVEGFR-2 were negatively linearly associated with blood platelet count and diuresis (Table 2).

### VEGF kinetics in plasma and urine samples

**Fig. 2:**
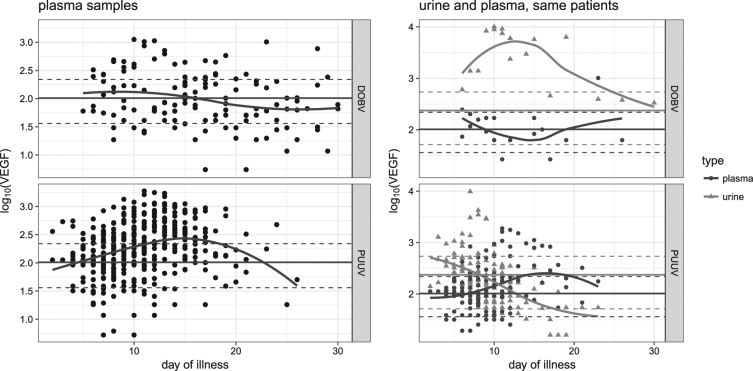
Kinetics of plasma VEGF and comparison of the kinetics in paired (plasma and urine) samples concomitantly collected; relation to different hantavirus species. Daily plasma kinetics (LEFT; DOBV [UP], PUUV [DOWN]). Concomitantly collected plasma and urine (RIGHT; DOBV [UP], PUUV [DOWN]) samples were available from 21 patients (19 infected with PUUV, 2 with DOBV). Levels of VEGF are shown as log_10_ pg/ml. The horizontal line represents mean levels in the control group; dashed lines indicate 95% confidence intervals. Each dot represents one measurement in a tested patient

Analysis of the daily kinetics of plasma VEGF showed significantly higher levels in HFRS patients than in the control group only between day 10 and day 15 of the illness (Fig. 2, left). Comparison between DOBV- and PUUV-infected patients showed that in DOBV infection, plasma VEGF levels were higher at the beginning of the disease (up to day 12), whereas in PUUV infection, plasma VEGF peaked later, around day 15 of the HFRS, i.e., at the time when patients usually enter the recovery stage of the disease. No significant difference was observed when VEGF kinetics were observed over time.

VEGF kinetics were also investigated in concomitant samples of plasma and urine. In DOBV-infected patients, plasma VEGF levels were normal almost all the time, but in urine they were considerably elevated (Fig. 2, right). However, for DOBV urine samples, statistical analysis was impossible because of the low number of available samples. However, in PUUV-infected patients, higher VEGF levels were detected in urine samples, with a peak in the first days of illness.

### sVEGFR-2 kinetics in plasma and urine samples

**Fig. 3:**
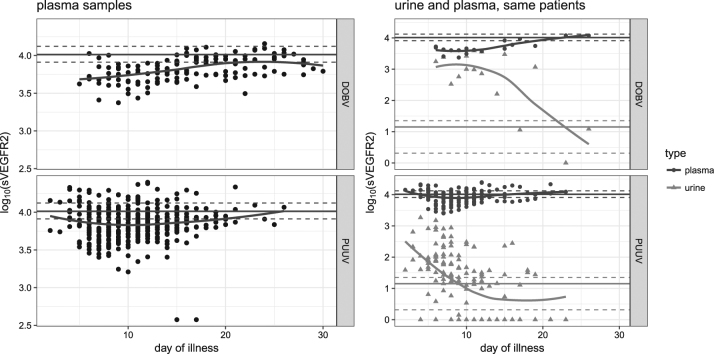
Kinetics of plasma sVEGFR-2 and comparison of the kinetics in paired plasma and urine samples concomitantly collected; relation to different hantavirus species. Daily plasma kinetics (LEFT; DOBV [UP], PUUV [DOWN]). Concomitantly collected plasma and urine (RIGHT; DOBV [UP], PUUV [DOWN]) samples were available from 21 patients (19 with PUUV, 2 with DOBV). Levels of VEGF are shown as log_10_ pg/ml. The horizontal line represents the mean level in the control group; dashed lines indicate 95% confidence intervals. Each dot represents one measurement in a tested patient

Investigation of sVEGFR-2 kinetics in plasma showed significantly decreased levels in HFRS patients at the beginning of the disease (up to day 12) in comparison with the control group (Fig. 3, left). However, levels of plasma sVEGFR-2 were higher in PUUV-infected patients than in DOBV-infected patients (*p* = 0.045).

Urine sVEGFR-2 levels were significantly higher in HFRS patients than in the control group during the first few days of illness (Fig. 3, right). In concomitantly obtained samples from HFRS patients, levels of sVEGFR-2 were lower in plasma but higher in urine samples (Fig. 3, right).

## Discussion

The vascular endothelium is a complex system that rapidly reacts and responds to the environment, changing from inactive to activated and back again. Several severe hemorrhagic syndromes, including HFRS, are characterized by excessive vascular permeability, microvascular thrombosis, and inflammation that results from endothelial cell dysfunction^30^. VEGF is a key regulator of normal angiogenesis, during which it promotes endothelial cell proliferation, differentiation, and migration. However, it also increases vascular permeability, mediates endothelium-dependent vasodilatation, and supports vascular survival by preventing endothelial apoptosis^24,31^.

In our study, we analyzed VEGF and sVEGFR-2 kinetics in HFRS patients and evaluated these biomarkers for the identification of individuals with progression to the severe form of the disease. In the assessment of VEGF and sVEGFR-2 kinetics in serial plasma samples from individual DOBV- and PUUV-infected patients, most plasma VEGF levels measured in HFRS patients were not significantly higher than those in the control group. However, based on the LOESS curve of VEGF dynamics, differences were observed in VEGF secretion in relation to virus species. In the PUUV-infected group, VEGF levels peaked between day 10 and day 15 of illness, at the time when patients usually enter the recovery stage of the disease and are discharged from hospital; however, in HFRS resulting from DOBV infection, no peak was seen (Fig. 2).

To analyze the value of VEGF as a biomarker for disease severity in HFRS patients, we compared acute plasma samples obtained from patients infected with DOBV and with PUUV in the first week of illness. In contrast to some studies in Hantaan- and DOBV-infected patients, where an association of elevated serum VEGF with the severe form of the disease has been reported^15,16,19–21^, no significant difference was observed in our study when comparing the severe and mild course of disease, regardless of the virus species. However, comparison of measured biomarker levels across different studies appears challenging, since we have shown that VEGF levels in serum and plasma samples from the same patient, collected on the same day, are significantly different (Wilcoxon test, *p* = 0.04). VEGF values in serum are higher than the corresponding values in plasma (237.8 vs. 112.9 pg/ml). It has also been reported previously that serum VEGF levels increase during clotting as a result of VEGF release from platelets and that plasma samples instead of serum represent free circulating VEGF more accurately^32,33^.

The most affected target organs in HFRS are the kidneys, and the infection often results in acute renal injury^26^; in the kidneys, VEGF and its receptors are expressed in glomerular podocytes and in tubular and peritubular epithelial cells^24,34^. The role of VEGF in normal renal physiology is essentially unknown, except that it is required for the growth and proliferation of glomerular and peritubular cells, and it is secreted in the urine directly from tubular cells under hypoxic stimulation. Thus, levels of urinary VEGF might be a unique indicator of renal hypoxia^24,34^. In DOBV-infected patients, we found an increase in urine VEGF almost throughout the hospitalization period. Comparison of VEGF dynamics in plasma and urine showed higher levels in urine. Moreover, in patients with chronic renal failure, VEGF secretion in urine increased as renal function decreased, suggesting increased VEGF secretion in residual nephrons during diffuse and continuous hypoxia ^34^. In our study, a large majority of patients had impaired kidney function with high creatinine values and low glomerular filtration rate, and 17.8% (13/73) of patients required dialysis treatment at least once during hospitalization.

With regard to the role of VEGF in hantavirus pathogenesis, an increase in plasma VEGF was associated with an increased platelet count, which is one of the first markers of clinical improvement. In contrast, the urine VEGF level was negatively associated with platelet count and diuresis. These results suggest a dual role of VEGF: first, local secretion of VEGF in the kidneys followed by renal impairment, and second, involvement in repair, as implied by higher levels of plasma VEGF and improvement of clinical parameters.

The effects of VEGF are mediated through two receptors, VEGFR1 and VEGFR-2, which soak up secreted VEGF and prevent engagement and activation of more distant endothelial cells. In vitro studies have shown increased vascular permeability upon treatment with VEGF, involving complex signaling processes through binding to VEGFR-2 and internalization of VE-cadherin^27^ or through activation of β3 integrin receptors, which are present on endothelial cells and are major receptors for pathogenic hantaviruses^13,28,35^. In DOBV-infected patients, the level of sVEGFR-2 was lower in initial plasma samples but higher in urine in comparison with the control group. The finding of normal plasma VEGF levels and lower plasma sVEGFR-2 levels suggests a substantial difference in the circulating active VEGF levels. A decrease of plasma sVEGFR-2 could be a potential mechanism for VEGF-directed systemic permeability, where VEGF contributes to capillary leak by not being inactivated by the receptor^31^. In the present study, the plasma level of sVEGFR-2 was negatively associated with viral load, but a positive linear association between plasma sVEGFR-2 levels and both platelet count and diuresis was recognized, with higher levels in the polyuric stage of the disease (8421 pg/ml; min-max: 4277–17,614 pg/ml). The urine levels of sVEGFR-2 in PUUV-infected patients decreased rapidly in a few days, but in DOBV-infected patients, they remained high up to day 17 of the illness. Increased sVEGFR-2 in the urine is probably a means of clearing the VEGF/sVEGFR-2 complex and may reflect the decreased sVEGFR-2 levels found in plasma. In urine, sVEGFR-2 levels were negatively associated with platelet count and diuresis in both virus species groups. Furthermore, patients with hemorrhagic manifestations had very high plasma (454 pg/ml; min-max: 106.5–1683 pg/ml) and urine (8034 pg/ml; min-max: 5574–12,395 pg/ml) VEGF levels and high levels of urine sVEGFR-2 (787 pg/ml; min-max: 6–2869 pg/ml).

Our study is the first study investigating daily secretions of VEGF and sVEGFR-2 in plasma and urine samples in patients infected with PUUV or DOBV. We have shown that VEGF plays a fine-tuning role in hantavirus pathogenesis; it is implicated in microvascular permeability at the beginning of the disease, possibly by decreasing receptor levels in the blood, and in the late phase it is involved in repair and remodeling of the vascular endothelium. Since VEGF acts in the near vicinity (within 0.5 mm) of its release, measuring local secretion of VEGF and sVEGFR-2 in urine might be useful biomarkers for identifying those HFRS patients who will progress to severe disease, but it requires further study.

## Materials and methods

### Patients

The study was performed on 73 hospitalized HFRS patients (58 males, 15 females), 58 infected with PUUV and 15 infected with DOBV. Diagnosis of HFRS was based on clinical findings (at least two of three: fever >38 °C, acute kidney injury, thrombocytopenia) and was confirmed serologically by an indirect immunofluorescence assay (IFA), enzyme-linked immunoassay IgM and IgG tests (ELISA) specific for DOBV and PUUV as well as with molecular one-step quantitative reverse transcription RT-PCR assay tests^11,36,37^. Acute DOBV or PUUV infection was confirmed molecularly in all patients included in the study.

After each patient was discharged from the hospital, a detailed medical chart was collected, and significant clinical and laboratory parameters were collected in order to grade disease severity (Supplementary Table S1). The criteria for severe HFRS were as follows: thrombocytopenia <50×10^9^/l and the need for dialysis; or thrombocytopenia <50×10^9^/l and the presence of >2 of the following: bleeding, oliguria/anuria, and levels of urea and/or creatinine at least 4× higher than the upper normal level. Patients who did not meet the above criteria were allocated to the mild disease category. Among the patients infected with PUUV, 15 were categorized as having severe disease, and 43 had mild disease. Among the 15 patients infected with DOBV, 6 fulfilled the criteria for severe disease.

To study the kinetics of secretion of VEGF and sVEGFR-2, 599 serial blood with EDTA and 182 serial urine samples were analyzed. Both blood and urine samples were collected in the morning and then centrifuged (plasma was removed from cells and stored separately), aliquoted and stored at −80 °C until further use. Plasma and urine aliquots were used to determine VEGF and VEGFR-2 concentrations, and cell aliquots were used for RNA isolation. In addition, plasma and serum samples were collected on the same day from 29 patients to compare VEGF levels in both sample types.

### Control group

The control group consisted of 51 healthy adult volunteers (32 males, 19 females) with an age distribution between 18 and 62 years (mean 37). Plasma (51) and urine samples (31) were concomitantly collected and prepared using the same protocol as for the patients.

### RNA extraction

Total RNA was extracted from whole blood samples with a TRIzol Plus RNA purification kit (ThermoFisher Scientific, MA, USA) in accordance with the manufacturer’s instructions.

### Virus genetic typing

The genetic typing of PUUV and DOBV was done with a multiplex real-time RT-PCR assay specifically targeting Slovenian DOBV and PUUV genetic lineages. The multiplex real-time RT-PCR assay targeted the DOBV M segment (97 bp) and PUUV S segment (186 bp) and was performed using primers DOB D (ACTTTAAGACAACCAATA), DOB L (GGGCAGTGTATTTATTCAG), PUU D (GGAGTAAGCTCTTCTGC), PUU L (ACATCATTTGAGGACAT) and probes DOB MGB (FAM-ACCACATTCTGCTTTGG-MGB-NFQ) and PUU MGB (VIC-AGACCAAAGCATTTATATG-MGB-NFQ). Real-time RT-PCR conditions were established for ABI7500 Fast (ThermoFisher Scientific, USA) with the following temperature protocol: 50 °C for 5 min, 95 °C for 20 s, followed by 45 cycles of 95 °C for 3 s, 55 °C for 30 s and 60 °C for 30 s. A total of 20 µl of reaction mix consisted of: 5 µl TaqMan^®^ Fast Virus 1-Step Master Mix, 0.6 µl of 50 µM primer DOB D, 0.4 of 50 µM primers DOB L, PUU D and PUU L, 0.3 µl of 20 µM of probes DOB MGB, PUU MGB, 7.6 µl MGB water (MolBio grade, Hamburg, Germany) and 5 µl of the extracted RNA.

### Viral load

Viral load in the whole blood samples was measured daily using a quantitative version of the multiplex RT-PCR assay described above. For standards, DNA fragments containing sequence information from Slovenian DOBV, PUUV and a control sequence (375 bp) were synthesized through gBlocks^®^ Gene Fragments (Integrated DNA Technologies, Coralville, Iowa). Standards were prepared according to the manufacturer’s instructions.

### VEGF

VEGF level was measured using the MILLIPLEX MAP Human Cytokine/Chemokine Magnetic Bead Panel (HCYTOMAG-60K; Merck Millipore, Darmstadt, Germany) according to the manufacturer’s instructions. The test was performed on a MagPix instrument (Merck Millipore), and the results were analyzed using Xponent Software 4.2 and the Milliplex Analyst program (Merck Millipore).

### sVEGF-R2

Soluble VEGFR-2 was measured using a Quantikine^®^ ELISA kit (R&D Systems, USA) according to the manufacturer’s instructions.

### Statistical analysis

The R environment was used for all statistical analyses. Smooth lines in graphs were calculated using the LOESS smoothing procedure (local polynomial regression fitting) in order to gain an impression of general time trends in the data. As most patients were not hospitalized for longer than 30 days post-onset of the disease, later measurements were omitted from the statistical analyses. In the control group, 95% confidence intervals were calculated using bootstrap (in order to avoid the influence of outliers). The bounds are plotted as dashed horizontal lines in the graphs. A confidence level of 0.05 was used in exploratory mixed-effect regression models (R package nlme) for comparisons between the viruses or levels of disease severity. In mixed-effect models for the assessment of associations between VEGF/sVEGFR-2 and clinical/laboratory parameters, a confidence level of 0.005 was considered significant (Bonferroni correction for 17 tests). The same confidence level was used in Mann–Whitney tests for pair-wise comparisons between patients and controls and for testing differences in initial samples (multiple comparisons). Only descriptive reports were made for comparison of a subgroup of DOBV patients with urine samples, since the patient group was too small to permit statistical evaluation.

### Disclaimer

The funders had no role in study design, data collection and analysis, decision to publish, or preparation of the manuscript.

## Supplementary information

Supplementary TableS1
