# Supplementary material for: Relationship between circulating vascular endothelial growth factor and its soluble receptor in patients with hemorrhagic fever with renal syndrome
Source: Emerg Microbes Infect. 2018 May 16;7:89. doi: 10.1038/s41426-018-0090-5 (PMC5953927; doi:10.1038/s41426-018-0090-5)
Supplement: Supplementary file 1 — Supplementary TableS1 [file 41426_2018_90_MOESM1_ESM.doc]

**Supplementary Table 1.** Clinical and laboratory characteristics of patients with HFRS included in the study. *^1^The most abnormal measurement for each parameter was selected. Normal values: platelet count (120–180 x 10^9^/L), white blood cell count (WBC: 4–10 x 10^9^/L), C-reactive protein (CRP: <5 μM/L), creatinine (44–97 μM/L), alanine transaminase (ALT: 0.74 μkat/L), aspartate transaminase (AST: 0.58 μkat/L), procalcitonin (< 0.5 µg/L), estimated glomerular filtration rate (eGFR >60 MDRD), D-dimer (<0.3 mg/L).*

|  | **DOBV** | | **PUUV** | | |
| --- | --- | --- | --- | --- | --- |
|  | severe  n=9 | mild  n=6 | severe  n=15 | mild  n=43 |  |
| Age (median, range) | 36 (18–81) | 39 (22–55) | 39 (22–61) | 39 (19–73) |  |
| Sex, n female/male | 2/7 | 0/6 | 3/12 | 10/33 |  |
| Days of hospital care  (median, range) | 41 (17–90) | 9 (7–29) | 11 (8–32) | 9 (5–17) |  |
| **CLINICAL SYMPTOMS** | | | | |  |
| Fever, n (%) | 8 (89) | 3 (50) | 15 (100) | 36 (84) |  |
| Headache, n (%) | 5 (56) | 1 (17) | 12 (80) | 31 (72) |  |
| Abdominal pain, n (%) | 8 (89) | 4 (67) | 9 (60) | 24 (56) |  |
| Nausea and vomiting, n (%) | 7 (78) | 5 (83) | 8 (53) | 23 (53) |  |
| Diarrhea, n (%) | 7 (78) | 4 (67) | 5 (33) | 11 (26) |  |
| Myopia, n (%) | 3 (33) | 1 (17) | 8 (53) | 13 (30) |  |
| Respiratory symptoms, n (%) | 2 (22) | 1 (17) | 4 (27) | 8 (19) |  |
| Dialysis, n (%) | 9 (100) | 0 | 4 (27) | 0 |  |
| Hemorrhagic manifestations, n (%) | 5 (56) | 2 (33) | 5 (33) | 6 (14) |  |
| **LABORATORY PARAMETERS^1^** | | | | |  |
| Min platelet, 10^9^/L  (median, range) | 33 (5–90) | 77 (22–113) | 46 (31–119) | 67 (20–253) |  |
| Min WBC, 10^9^/L  (median, range) | 6 (4.7–8.8) | 3.9 (2.6–6.2) | 7.4 (6.1–10.3) | 5.6 (2.9–12.8) |  |
| Max CRP, mg/L  (median, range) | 115.5 (73–436) | 50 (33–105) | 96 (5–394) | 95 (13–270) |  |
| Max creatinine, μM/L  (median, range) | 817 (418–975) | 483 (324–538) | 627 (105–1023) | 194.5 (72–702) |  |
| Max ALT, μkat/L  (median, range) | 1.5 (0.7–15.6) | 1.5 (0.6–3.2) | 1.2 (0.5–3.6) | 1.0 (0.6–6.3) |  |
| Max AST, μkat/L  (median, range) | 2.9 (0.6–20.8) | 1.2 (0.8–2.5) | 1.1 (0.5–1.8) | 1.0 (0.4–4.4) |  |
| Max procalcitonin, µg/L  (median, range) | 4 (1.4–23) | 3 (0.8–4) | 2.1 (1.1–9.9) | 1.6 (0.4–6.9) |  |
| Min eGFR, MDRD  (median, range) | 7.5 (5–12) | 12 (10–19) | 8 (5–60) | 24 (7 – 60) |  |
| Max D-dimer, mg/L  (median, range) | 5.5 (1–43.4) | 3.1 (1–5.3) | 6 (4.9–23.1) | 4.5 (0.9–36.4) |  |
